# Pulmonary and Physical Function Limitations in Aging Men with and without HIV from the Multicenter AIDS Cohort Study

**DOI:** 10.1093/geroni/igab046.2333

**Published:** 2021-12-17

**Authors:** Mona Abdo, Ken Kunisaki, Valentina Stosor, Gypsyamber D'Souza, Madiha Abdel-Maksoud, Todd Brown, Samantha MaWhinney, Kristine Erlandson

**Affiliations:** 1 Colorado School of Public Health, Aurora, Colorado, United States; 2 University of Minnesota Medical School, Minneapolis, Minnesota, United States; 3 Northwestern University Feinberg School of Medicine, Chicago, Illinois, United States; 4 Johns Hopkins Bloomberg School of Public Health, Baltimore, Maryland, United States; 5 Johns Hopkins School of Medicine, Baltimore, Maryland, United States; 6 Colorado School of Medicine, Aurora, Colorado, United States

## Abstract

We sought to determine effects of age, HIV serostatus, and smoking on the associations between pulmonary function and physical function impairments using Multicenter AIDS Cohort Study data. Associations between physical function outcomes gait speed (m/sec) and grip strength (kg) with normalized pulmonary function tests (diffusion capacity for carbon monoxide (DLCO, n=1,048) and forced expiratory volume in one second (FEV1, n=1,029)) were examined. Adjusted mixed-effects models included interaction terms to assess effect modification. 574(55%) were HIV+, with median age 57(IQR=48,64) and mean cumulative smoking pack-years 12.2(SD=19.0). 349(33%) had impaired DLCO (<80% of predicted) and 130(13%) had impaired FEV1 (<80% of predicted). Participants with impaired DLCO had weaker grip strength than those with normal DLCO (estimate= -3.5[95% CI=-4.6,-2.4]kg; p<0.001). Participants with impaired DLCO had slower gait speed than those with normal DLCO (estimate= -0.04[95% CI= -0.06,-0.02]m/sec; p=0.002). Age modified the DLCO effect on gait (p-interaction=0.01) but not grip (p-interaction=0.09). The association between decreased DLCO and slower gait was more pronounced in older participants. Smoking or HIV serostatus did not significantly modify the DLCO effect on gait (all p-interaction≥0.14) or grip (p-interaction=0.74, p-interaction=0.058, respectively). As with DLCO, participants with impaired FEV1 had weaker grip strength (estimate=-3.0[95% CI= -4.7,-1.3]kg; p<0.001) than those with normal FEV1. FEV1 was not associated with gait speed(p=0.98). Age, HIV serostatus or smoking did not modify the associations between FEV1 and gait speed or grip strength (all p-interaction>0.05). Associations between lower DLCO/FEV1 and decreased physical function suggest that interventions to improve pulmonary function may also preserve physical function with aging.

